# Building information modelling review with potential applications in tunnel engineering of China

**DOI:** 10.1098/rsos.170174

**Published:** 2017-08-02

**Authors:** Weihong Zhou, Haiyang Qin, Junling Qiu, Haobo Fan, Jinxing Lai, Ke Wang, Lixin Wang

**Affiliations:** 1 School of Construction Management and Real Estate, Chongqing University, Chongqing 400044, People's Republic of China; 2 School of Highway, Chang'an University, Xi'an 710064, People's Republic of China; 3 China Railway First Survey and Design Institute Group Co., Ltd., Xi'an 710043, People's Republic of China

**Keywords:** tunnel engineering, building information modelling, engineering management, China

## Abstract

Building information modelling (BIM) can be applied to tunnel engineering to address a number of problems, including complex structure, extensive design, long construction cycle and increased security risks. To promote the development of tunnel engineering in China, this paper combines actual cases, including the Xingu mountain tunnel and the Shigu Mountain tunnel, to systematically analyse BIM applications in tunnel engineering in China. The results indicate that BIM technology in tunnel engineering is currently mainly applied during the design stage rather than during construction and operation stages. The application of BIM technology in tunnel engineering covers many problems, such as a lack of standards, incompatibility of different software, disorganized management, complex combination with GIS (Geographic Information System), low utilization rate and poor awareness. In this study, through summary of related research results and engineering cases, suggestions are introduced and an outlook for the BIM application in tunnel engineering in China is presented, which provides guidance for design optimization, construction standards and later operation maintenance.

## 1. Introduction

**Figure 1.**
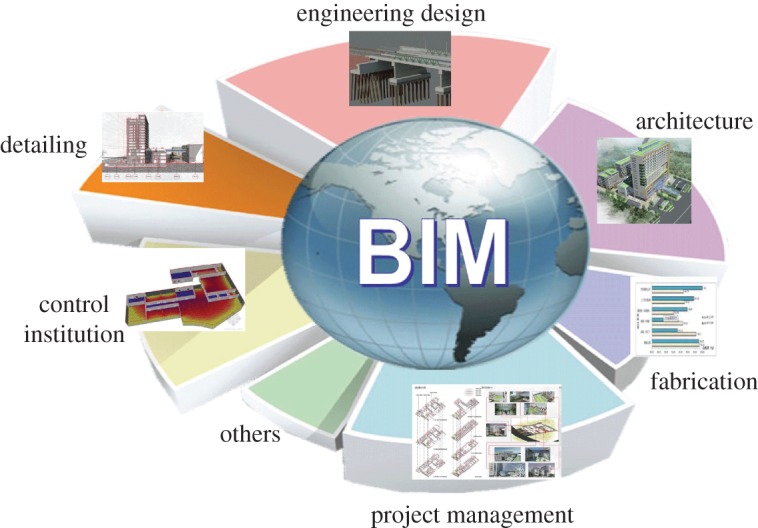
Application of BIM in various fields of architecture.

Building information modelling (BIM) technology was first proposed by Dr Chuck Eastman from Georgia Institute of Technology in the United States in 1975 [1]. Later, Jerry Laiserin and others supplemented and standardized this conception, further popularizing it [2]. In 2002, Phil Bernstein publicized related software by adopting BIM conception for the first time [2]. Thereafter, General Services Administration (GSA) launched a 3D-4D-BIM programme in 2003 and subsequently presented a series of BIM guiding suggestions [2]. The United States Army Corps of Engineers (USACE) formulated and issued a 15-year (2006–2020) BIM development roadmap in 2006. The National Institute of Building Sciences formulated National BIM Standard (NBIMS) in 2007, and research into BIM application was conducted by the Building SMART Alliance (BSA) [3]. By the end of 2008, the BSA had obtained a series of standards, including the Industry Foundation Classes (IFC) standard, the United States National CAD Standard and the Journal of Building Information Modeling (JBIM) standard [4]. Wisconsin became the first state in America to adopt the requirement that new large-scale public construction projects should include BIM. In 2009, the Japan Ministry of Land, Infrastructure, Transport, and Tourism started to introduce BIM technology and, now, BIM has been applied to the whole of Japan [5]. Additionally, many government agencies in Europe and South Korea are also devoted to formulating application standards of BIM, all of which will lead to an unprecedented revolution in architecture (figure 1) [6–11].

BIM technology started relatively late in China [12] and knowledge of this technology has gradually spread throughout civil engineering [13]. Only some large design enterprises attempt to apply BIM technology into project design. For instance, in 2005 the Shenyang Architecture Design Institute began to apply Revit software in collaborative design, and in 2010 the China Architecture Design & Research Group (CAG) applied BIM to collaboratively design the Dunhuang Tourist Center project. The majority of small and medium-sized construction companies have not yet integrated the BIM technology, and they still employ traditional auto-CAD (computer-aided design) technology for graphic design [14,15]. Recently, research, development and application of BIM have become an important subject in China [16]. In January 2012, a notice on Issuing the Formulation and Revision Plan for Engineering Construction Standards in 2012 was released by the Ministry of Housing and Urban-Rural Development of the PRC (The People's Republic of China), signifying that formulation for BIM standards in China had officially started. So far, a series of BIM standards have been issued. In October 2013, the Engineering Quality Safety Supervising Department under the Ministry of Housing and Urban-Rural Development of the PRC issued the Guidance for Promoting the Application of BIM Technology in Building Field, which defined the development goal of BIM in China [17].

Compared with common architecture projects, tunnel engineering features more complex geological conditions, more construction quantities, more unknown factors and more significant resource allocation during construction. All of this resulted in the advantages of BIM in the tunnel industry being greater than in the construction industry; therefore, the tunnel industry introduced a more urgent requirement for BIM technology. However, the informatization level of tunnel engineering in China still remains low; although manual drawing has already been replaced by CAD and the efficiency of a tunnel engineering design has already been significantly improved, it is still a concentration-deviating management mode, which is significantly different from the concentrated resource management requirement of BIM [18]. Tunnel engineering, in contrast with industrial and civil building engineering, has its own special characteristics. Firstly, tunnel engineering is characterized by the zonal distribution with length varying from several hundred metres to several kilometres, and this is entirely different from industrial and civil building, which is situated in centralized areas [19]. Secondly, the tunnel is usually located underground and closely related to the surrounding geology. Therefore, the accuracy of geological survey greatly affects the quality of tunnel design. Moreover, contents and attributes in industrial and civil building engineering is different to tunnel engineering so that existing building code classifications cannot properly cover tunnel engineering; therefore, the BIM standard cannot be directly applied to tunnel engineering, and the BIM technical route for industrial and civil building cannot be absolutely followed by the application of BIM technology in tunnel engineering [20,21]. To facilitate the development of BIM technology in tunnel engineering in China, it is necessary to systematically analyse its problems and corresponding research results, which can provide certain experiences for construction relevant in tunnel engineering.

## 2. Building information modelling technology

### 2.1. Characteristics

**Figure 2.**
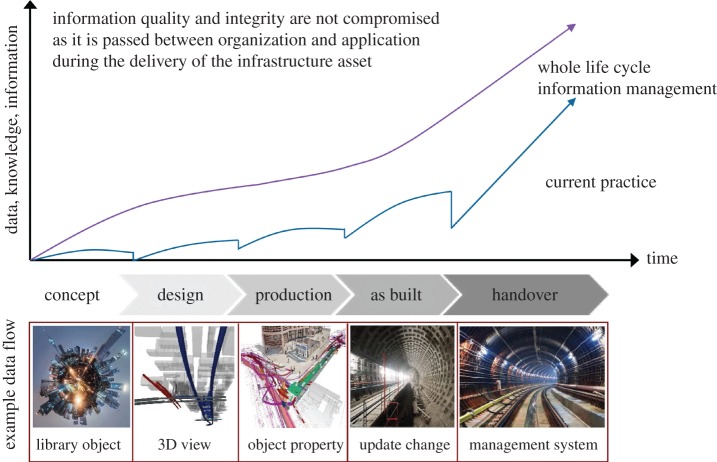
Effect of BIM technology on information transmission.

BIM integrates both digitalization and informatization function of building models, and nearly covers all the information of construction projects [6,22]. Information files of the entire model are delivered by the designer to the constructor and ultimately saved by the operator after accomplishment of the entire construction, which is convenient for subsequent maintenance and management [23,24]. During the whole process, different units of designer, constructor and operator can all apply and modify the plans [25]. The best advantage of BIM technology is its unique approach of saving the project information in a unified way, so that this building information model can be applied to the full life cycle of the project [26,27]. The impact of the traditional information management mode and the BIM-based information management mode on the efficiency of information transfer is shown in figure 2.

### 2.2. Building information modelling in tunnel engineering

BIM in tunnel engineering strives to introduce BIM technology into tunnel engineering, so that the design, construction and operation stages of a tunnel project can be presented in the form of an informatizational model, and a 3D visual experience of the tunnel can even be enjoyed just before construction [28,29]. Furthermore, many operations that have once been difficult to accomplish in the later period can then be flexibly achieved, such as the refinement of model design, the safety of construction and systematic operation [30], each of which definitely contributes to the improvement of the design in terms of quality and efficiency. However, costs and intensity of work can also be reduced, and the cooperation among different units can be enhanced [31].

More obstacles exist in the implementation of BIM-based tunnel engineering: the first one is the selection of suitable software platforms. BIM technology for the architecture industry cannot be directly applied to the tunnel industry, because previous BIM-based software almost only caters to the design of the architecture industry and is not in accord with tunnel engineering standards and software. Compared with the architecture industry, tunnel engineering features more complexity, including complex geological conditions due to uneven terrain, large project scale and unpredictable factors, such as water gushing and fragile surrounding rock [32–34]. Therefore, the advantages of BIM technology applied in tunnel engineering are more obvious because tunnel engineering is confronted with various difficult problems, including unknown factors, longer construction cycle, limited construction space, higher requirements for operation and maintenance, and more severe consequence of fire and traffic accidents [35,36]. For example, 4D simulation of real construction and collision detection can be performed to discover unknown difficulties after the accomplishment of BIM-model design. The advantages that BIM technology introduces in the entire life cycle of project management are more obvious due to the features of the construction cycle of the tunnel project. Owing to the very severe consequences of fire accidents in tunnels, the interactive operation between BIM technology and severe game platforms can be applied to address the data shortage existing in the simulation method in observing personal escape behaviour in a tunnel fire accident [37].

The implementation process of BIM technology-based tunnel engineering is as follows: (i) A 3D geological model is created and combined with geological information. (ii) A tunnel route is selected based on the geological model. (iii) Sections of the route are cut to conduct sketch design and parametric design according to the actual environment. (iv) CATIA and other modelling software packages are applied to generate a 3D tunnel entity by various operations of stretch, multi-sections, shifting and array. (v) Information is added to the tunnel model, including attributes, description attachment, parameter setting, external links and database storage.

**Figure 3.**
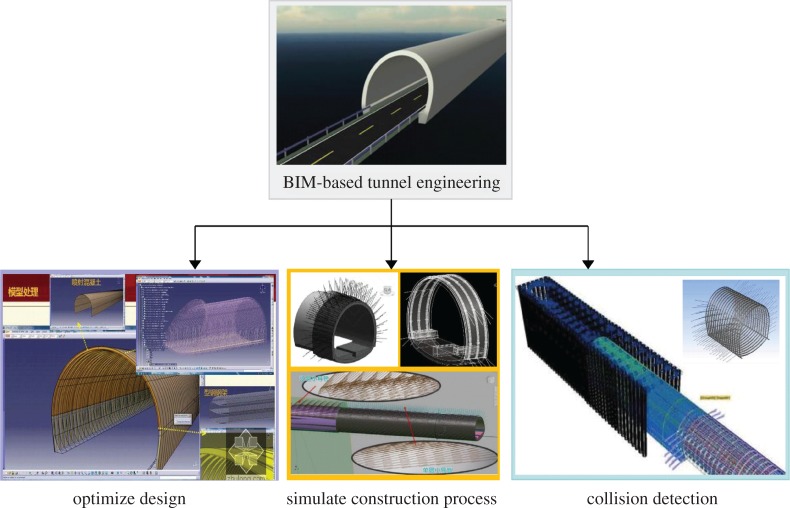
Some functions of BIM in tunnel engineering.

A BIM-based tunnel project can achieve obvious benefits. Taking the metro tunnel as an example, BIM can be applied to reach extra functions such as detailed design, collision detection, 2D and 3D drawing, and statistics of quantities during the design stage [38–43]. During the construction stage, BIM can also be applied to accomplish construction simulation, resource allocation and construction setting. During the later period, the BIM model can be applied to operation management and emergency rescue work of the metro project [44–51], as shown in figure 3.

## 3. Application results of building information modelling in tunnels

### 3.1. Case statistics

The case statistics were conducted about BIM application in tunnel engineering in China, and the results are shown in table 1.

**Table 1.** Statistics results of BIM application in tunnel projects of China.

project	features	stages of BIM application	design unit	result	problems
Xingu Mountain tunnel [31]	58 types of cross-section, vertically crossing above the existing railway tunnel	the whole design process from feasibility design to drawing design	China railway Siyuan survey and design group	multi-professional integration of 8199 m in Navisworks, 4D simulation of construction process	have no software for generating 3D geological model, have no BIM standard, compatibility issues of BIM software
Shanghai Yuejiang tunnel [47]	complex underground pipeline, requirements of land subsidence is strict	feasibility design, construction progress detection, collision detection, engineering quantity statistics	Shanghai urban construction design and research institute	reduce the danger caused by changing pipeline route, improve the coordination efficiency between various projects	failure to apply BIM in all stages, basically apply building BIM software
Qingliang Mountain tunnel [48]	typical mountain tunnel, complex geology, larger tunnel curve	design stage, construction stage, engineering budget, aided drawing	China railway Yiyuan survey and design group	optimize design quality, improve management level	have problems in software specialization, localization, standardization and compatibility
NO.2 Benzhong Moutain tunnel [49]	located in Tibet, China, undulating terrain, covered with frozen soil, an elevation of about 1700 m	design stage, 4D construction simulation	China railway Eryuan engineering group	conduct key measures on weak parts, reduce the occurrence of dangerous accidents	BIM application scope is small, mainly used for auxiliary design
Metro Line 7 in Beijing [50]	complex underground pipeline, intensive ground building	preliminary planning, visual design, automatic collision detection, construction drawing design	Beijing general municipal engineering design and research institute	analysis and calculation of daily passenger flow, designers and managers to share resources	mainly rely on REVIT software
Shigu Mountain tunnel [51]	broken loess plateau, poor geological condition	model design, collision detection, aided drawing	China railway Eryuan survey and design group	improve design precision, shorten construction cycle, ensure construction quantity	BIM technology is mainly applied in design stage, does not exist in all stages of project

Table 1 indicates that the application of BIM in tunnel engineering in China mainly concentrates on design and collision-detection stages of a project, and this BIM model cannot be applied for the entire period of a project because certain incompatibility exists among different units of designer software, construction software and operator software. At present, the BIM application in tunnel engineering is still at its initial stage in China; in the imminent future, this technology will gradually extend to other fields such as the construction of mountain tunnels, subways and municipal tunnels. The combination of tunnel engineering with BIM technology will be the general trend along with higher requirements for environmental protection and informatization.

Compared with the application of BIM technology in the architecture industry, the two most severe difficulties in the tunnel industry are the generation of geological BIM of the complex mountain and the incompatibility between software in different stages. At present, the software applied in tunnel BIM mainly caters to the architecture and machinery industry; consequently, they are not proficient in tunnel engineering, and the majority of them are mainly applied in the exploration stage. Therefore, in the following two cases, the generation of a 3D geological BIM (Xingu Mountain tunnel) and the application of BIM technology in the entire period of design, construction and operation stages (Shigu Mountain tunnel) will be briefly analysed.

### 3.2. Case study 1: the Xingu Mountain tunnel

The case study of the Xingu Mountain tunnel emphasizes the creation of a 3D geological model. At present, there is no software that could meet all the functions required by a BIM model in the tunnel field in China. In this case, a combination of Inventor, Civil 3D, Google Earth and other geological software was applied to complete all this function during the design of a BIM model for the Xingu mountain tunnel. Firstly, the profile of the mountain was generated by applying Civil 3D software. Then, Google Earth data were imported, and the next step was to complete the generation of the entire mountain model by integration with the corresponding geological software. Finally, the tunnel BIM model was established on this basis.

#### 3.2.1. Project information

**Figure 4.**
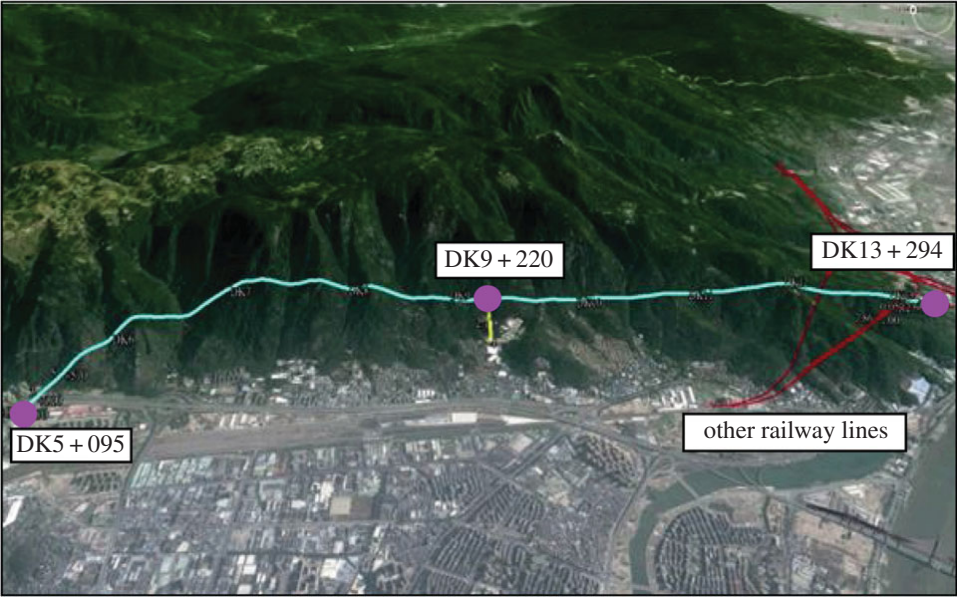
Topographic map of Xingu Mountain tunnel.

The Xingu Mountain tunnel passes through the Gushan Mountain scenic area of Fuzhou city, and its entrance is located at the east side of Dongshan village of Fuzhou city, about 400 m away from Dongshan village, passing the south 3rd ring and airport expressway at, respectively, DK5 + 205 and DK5 + 230. The exit is located on the slope at the north side of Dongshan village of Fuzhou city, vertically crossing above the existing Wenzhou–Fuzhou and Fuzhou–Xiamen railway tunnel at DK12 + 189, DK13 + 009 and DK13 + 319. The starting and ending mileage of the tunnel is DK5 + 095 and DK13 + 294, with a total length of 8199 m. The tunnel has one auxiliary transverse hole for construction, which is located at the right side of the tunnel, and intersects with the tunnel at DK9 + 220, with an included angle of 90°, and a total length of 400 m. The topographic map of the Xingu Mountain tunnel is shown in figure 4.

#### 3.2.2. Development and challenges

##### 3.2.2.1. External challenge: development status of BIM in China

On the one hand, BIM, as a recently emerging technology, requires the coordination of different units, specialties and construction stages, and is strongly dependent on its accessory software. On the other hand, BIM technology started relatively late in China, and software development still remains insufficient in which the BIM technology in China is available during the design stage of projects. Moreover, the application of tunnel BIM technology is more complicated than that of building BIM applications. Prior to this, only a few BIM technology-based tunnel projects were reported in China without ready-made software for the generation of a 3D geological model.

##### 3.2.2.2. Internal challenge: complicated topography of the Xingu Mountain tunnel

The Xingu Mountain tunnel is characterized by complicated sections and large span, with a total length of 8199 m, involving dozens of section types and a large quantity of engineering. Therefore, reasonable and orderly division of the entire work should be accomplished before the application of BIM technology in the design stage.

##### 3.2.2.3. BIM model establishment

BIM design of the Xingu Mountain tunnel mainly adopted the IDS package and architecture software such as Inventor as core software, and the following methods were adopted to build a 3D geological model of the Xingu Mountain tunnel.

**Figure 5.**
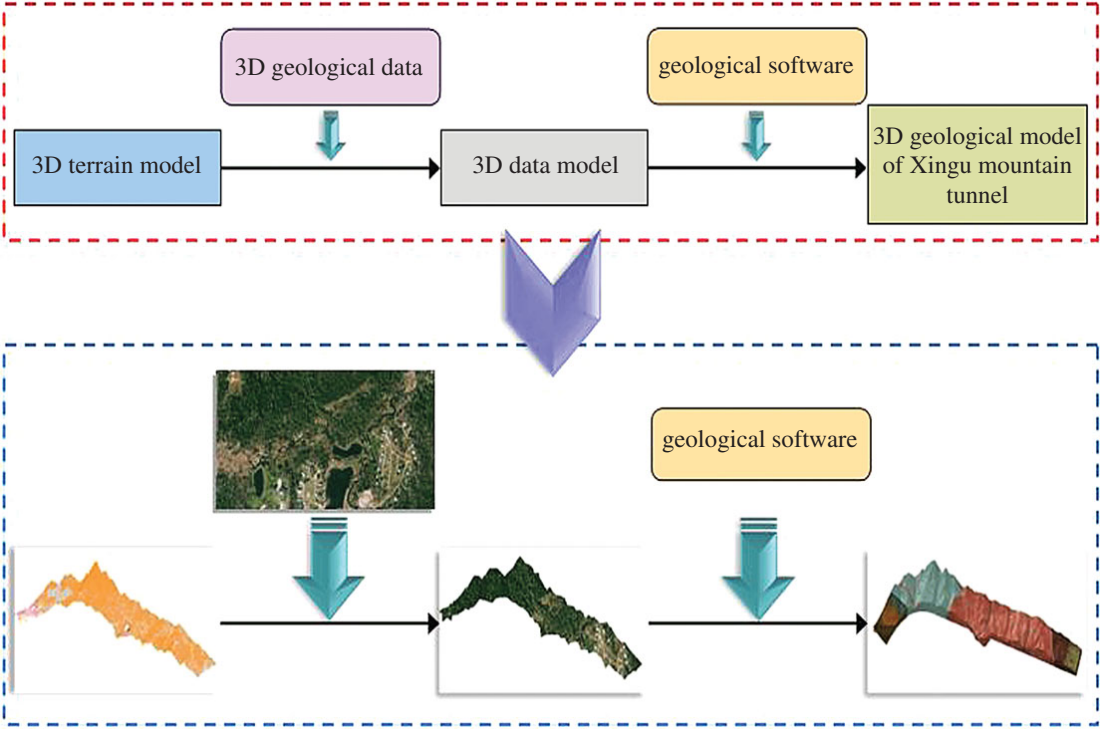
Steps for generating 3D geological model of Xingu Mountain tunnel.

(a) Civil 3D software was applied to build a 3D model of no-data information, and 3D geological data extracted by Google Earth were combined to generate a 3D data model.
(b) On the basis of this 3D data model, geological software was combined for professional geological design, to generate a 3D geological model of the Xingu Mountain tunnel. The specific steps for generating a 3D geological model of the Xingu Mountain tunnel are shown in figure 5.

With regard to the design of the tunnel structure, Inventor software was applied to build the material library corresponding to anchor bolt and steel arch. For the design of tunnel sections, the corresponding parameters were applied for control.

After completion of the model, 4D simulation was conducted for tunnel construction in Navisworks.

##### 3.2.2.4. Discussion

**Figure 6.**
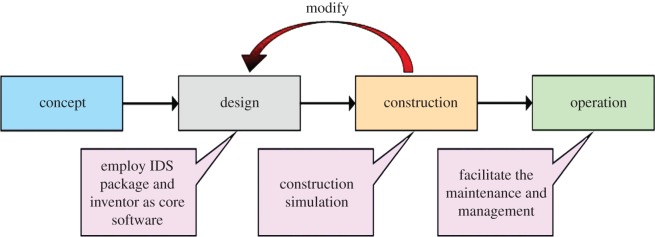
Implementation of BIM technology in Xingu Mountain tunnel.

BIM technology was involved during the stages of planning, design, construction and final operation of the Xingu Mountain tunnel. However, many defects still exist among various functions due to the unprofessional software of CATIA, such as the design of bolt attributes being too rough and the 2-D drawing being far too unprofessional. At present, BIM technology has been mainly applied during the design stage, and it was difficult to apply it to all stages. Therefore, the BIM application in this project developed a solid foundation for the development of BIM technology. The implementation process of BIM technology in the Xingu Mountain tunnel project is shown in figure 6.

### 3.3. Case study 2: Shigu Mountain tunnel

In the case study of the BIM model of the Shigu Mountain tunnel, the generation of the BIM model and its application during design, construction and operation stages are analysed in detail, while the accomplished BIM model cannot be applied in the entire period of a tunnel project because there is severe incompatibility among different units of design, construction and operation stages in the tunnel field. To solve this problem, CATIA software was firstly introduced to the tunnel field to try to build up the BIM model of the Shigu Mountain tunnel, and the result proves that CATIA software can be applied to meet the demands of information-transferring function, collision-probing function, quantity-calculating function and construction-simulating function. However, CATIA software is mainly aimed at the mechanical and aerospace field, and is not professional for the tunnel field; therefore, there are still many defects and large gaps to be improved, such as the low quality in detailed design.

#### 3.3.1. Project information

As a double track tunnel, the Shigu Mountain tunnel is located at a broken loess plateau at the south bank of the Weihe river of Baoji city, with the starting and ending mileage of DK639 + 430 and DK643 + 760, and a total length of 4330 m. The surface elevation of the tunnel is 624–766 m, and the scope of depth is 3–133 m. It is mainly in cultivated land, with dense vegetation. As with one of the high-risk tunnels of the Baoji-Lanzhou Railway, this tunnel passes through three rivers, which are characterized by complicated geological environments and extremely poor surrounding rock conditions.

#### 3.3.2. Planning and design

As there is no professional BIM software that can be applied during the entire period of a tunnel project, and a series of incompatibility problems exists among software of different stages, CATIA software was first introduced to build a tunnel BIM model in the Shigu Mountain project to solve these problems.

##### 3.3.2.1. 3D geological model

The 3D geological model was generated via the surface-meshing function of CATIA software; then, the geological layer combined with the mapping data were established, and vertical and cross-sections were selected. Next, the geological entity in combination with the sketching function was extruded; finally, the ground surface, formation face section and extrusion entity were employed to generate the geologic body.

##### 3.3.2.2. Skeleton inheritance

Based on a 3D geological model and 3D line model, 3D lines were inherited and extracted according to factors of geology, section and construction method. Then, the secondary skeleton of the tunnel was obtained.

##### 3.3.2.3. Parameter setting

Some factors including lining form, design custom, construction method and process, and information demand were all taken into consideration for parameter setting, e.g. parameters of profile, rebar, steel frame and joint, construction method, fore-poling and anchor bolt are set for the lining section of the V-level tunnel of the Shigu Mountain tunnel.

##### 3.3.2.4. Sketching

Combined with the demands of model building, and based on the requirements of clearance, gauge and speed in tunnel engineering, the sketch-design function of CATIA was adopted to draw a corresponding sketch of the profile.

##### 3.3.2.5. Model building

Considering that sketch-drawing was combined with parametric, the functions of CATIA, including stretch, multi-sections, shifting and engineering array, were used to generate 3D solids.

##### 3.3.2.6. Information addition

Information is the key aspect of the BIM model. A 3D model without information cannot be called a BIM model. The whole information of the BIM model mainly includes geometrical information and non-geometric information. Additional information applied to the Shigu Mountain tunnel mainly includes attributes, description addition, parameter setting, external links and database storage.

##### 3.3.2.7. Quantity calculation

The CATIA function, including parametric calculation and engineering drawing, was adopted for the Shigu Mountain tunnel to complete 2D quantity statistics. Furthermore, the 3D measurement function of CATIA was applied in the portal section to complete 3D quantity statistics.

##### 3.3.2.8. 2D drawing output

**Figure 7.**
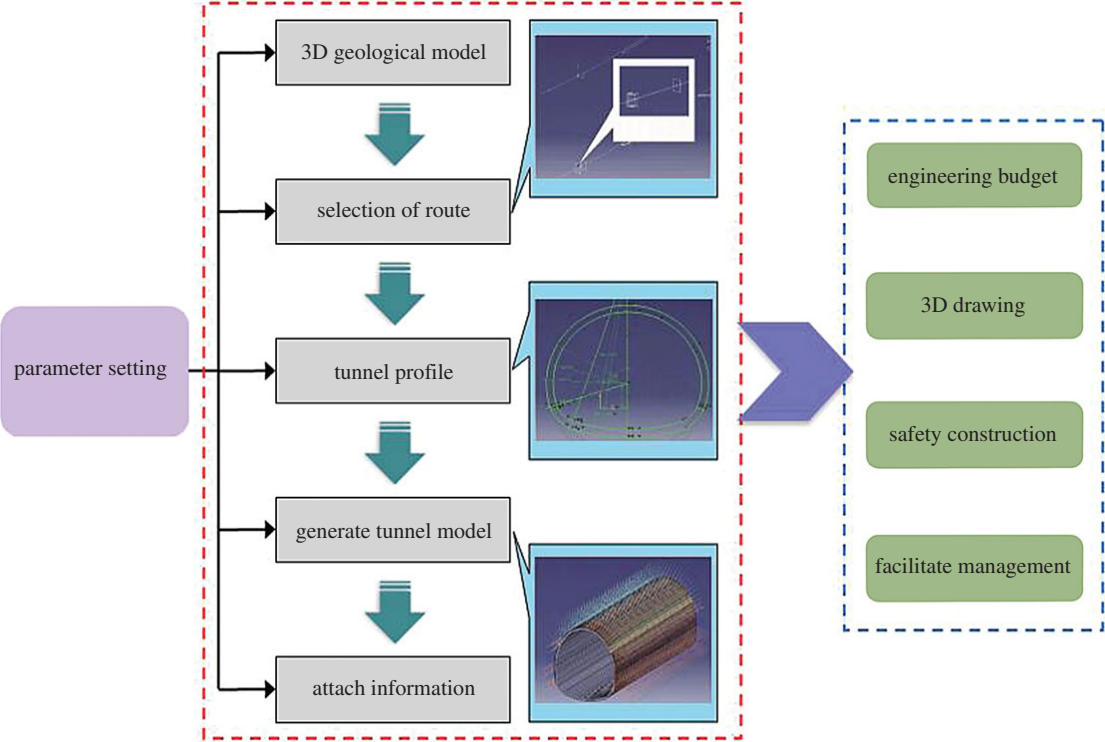
Application of BIM in Shigu Mountain tunnel.

In this project, some functions, including associated parameter, drawing, dimensional projection and marking, were adopted to realize the linkage and output of the 3D model and 2D engineering drawing. The application of the BIM model in the planning and design stage of the Shigu Mountain tunnel is shown in figure 7.

##### 3.3.2.9. Construction

Requirements for fineness and the amount of information in the construction stage differ from those during the design stage; thus, further processing should be made to design the BIM model before its application during the construction stage. The processing work during this stage mainly includes model splitting, resource allocation and construction setting.

##### 3.3.2.10. Model splitting

The BIM model from the design stage should be further split to satisfy the demand in the construction stage according to series factors such as construction division, construction arrangement and construction method. BIM application during the construction stage should be provided with personnel, machines, tools and other relevant resources according to construction conditions, to realize information management and simulation during the construction process.

##### 3.3.2.11. Construction setting

During the construction stage, DELMIA software of the Dassault series was mainly adopted for construction simulation and organization management. The software, in the form of Gantt chart, presents the process arrangement, connection, duration and other construction factors of the tunnel, further realizing the coordination and evaluation of various construction processes of the tunnel via programme evaluation and technique review, to achieve the goal of reasonable arrangement of labour resource, material resource, time and capital.

##### 3.3.2.12. Operation

During the operation stage, 3DVIA software of the Dassault series was applied for displaying the BIM models during design and construction stages, and the physical information, including spatial location information, structure, decoration, equipment and facilities. In the BIM model, information is displayed, processed and saved, to provide technical support for daily management during the operation stage. The emergency-saving case was simulated in the Shigu Mountain tunnel, such as ventilation organization and personnel evacuation.

##### 3.3.2.13. Discussion

Based on the Shigu Mountain tunnel project of the Baoji–Lanzhou railway, an exploration for a solution of BIM application in the whole life cycle of the railway tunnel can obtain the following achievements.

The successful BIM application, including 3D visualized modelling, information integration in stages of planning, design, construction and operation, drives the transformation of all stages from 2D to 3D, 4D and 5D, and from extensive orientation to elaborate orientation.

The 3D design improves the accuracy of the design, and achieves the optimization of the scheme. The 3D construction realizes the dynamic simulation, informatization management and engineering quantity statistics, to reduce risk and cost, and ensure the construction period. The 3D operation realizes elaborate and informationized asset management of both operation and maintenance, which provides effective information support for later maintenance and exercise.

The BIM application in the whole life cycle of tunnel engineering can provide technological support to solve problems of information chain breakage, extensive construction process and inspecting collisions, which can optimize design and control construction process as well as improve operation management.

### 3.4. Results and analyses

Through analysing cases of the Xingu Mountain tunnel and the Shigu Mountain tunnel, functions of BIM during different stages have been concluded as follows.

The design stage includes the establishment of a 3D model for overall structure and parameters design of cross-section, bolt and steel arch. A 3D model was established based on factors such as geological data, 3D modelling software, GIS and other geological and landform software. Parameter selection for cross-section and bolt mainly relied on the matching between modelling and material library built by CATIA, Inventor and other software.

During the construction stage, personnel, machines, equipment and relevant resources are allocated according to construction organization. The 4D simulation for construction and informatization of management are implemented. A dynamic construction simulation conducted before construction is helpful to decrease potential dangers during tunnelling. Furthermore, the construction cost can be significantly reduced.

During the operation stage, the display, processing and storage of spatial position and physical information in the BIM model are dynamically simulated, including structure, decoration, equipment, and facility, and emergencies such as fire disaster and traffic accident. It formulates corresponding schemes for ventilation and personnel evacuation, which have provided technology support for routine management.

**Figure 8.**
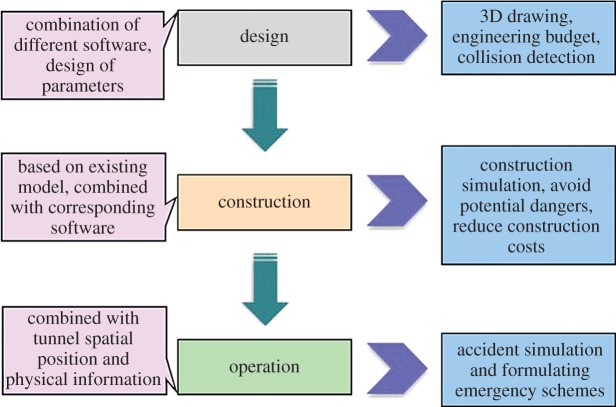
Role of BIM in the tunnel project.

BIM exists during all the stages of tunnel planning, design, construction and operation (figure 8). In the process, different units all can apply and modify models, in which communication and coordination among units are enhanced, production costs are reduced, construction quality is ensured and construction efficiency is improved. Therefore, introducing BIM technology into tunnel engineering has profound significance.

## 4. Problems and suggestions for building information modelling-based tunnel engineering

### 4.1. Problems

#### 4.1.1. Standard problem

BIM technology is based on mutual cooperation, which requires coordination among different software packages of modelling, drawing and simulation. Owing to different manufacturers, data exchange remains difficult. Many developed countries have formulated an appropriate standard system [52]. In China, due to the time of introducing BIM technology being rather short, only some basic studies have been conducted for BIM standards, and the localization is relatively low [53].

Problems of BIM-based tunnel engineering in China are influenced by many factors, which include the disunity of the system, the neglect of the BIM legal system and the lack of relevant personnel [54]. Realization of the BIM standard is closely related to the solution for all these problems, and any neglect could lead to failure in standard establishment.

#### 4.1.2. Platform problem

Currently, most companies in China mainly depend on foreign BIM software and the localization is relatively low. BIM software fails to consider the topographic condition, application mode and regulation requirement of China. Therefore, the secondary exploration about the software is undoubtedly necessary when BIM software is coordinated with other software packages [55], e.g. the unit system, interface expression, drawing regulation and construction regulation of REVIT (developed by the USA) are different to those in China. Although vast differences exist in reality, the BIM software has been welcomed due to the complete and comprehensive functions.

The geological conditions of the architecture industry are relatively simple; therefore, the existing BIM software can meet the requirements. However, as for tunnels, especially for mountain tunnels, existing geological software is unable to build corresponding models due to the features of complex geology and large span of tunnels. Furthermore, most existing geological software has incompatibility problems with other BIM designing software. Therefore, at present, the BIM application in tunnel engineering can only be based on the architectural BIM design software, which could generate the inconformity of application in platforms such as AutoDesk, Dassault or Bently. Different platforms would result in difficulties in model data exchange.

#### 4.1.3. Management mode

The DBB (design–bid–building) mode still remains the major building mode for tunnel engineering in China, limiting the communication among different units, hindering information flow and restraining the development of BIM for tunnel engineering. Currently, only special projects of tunnel engineering employ BIM technology. For general tunnel engineering, BIM technology is only applied during the design stage; there is still a large gap to meet the requirements of BIM technology. Research shows that the advantages of BIM could be amplified if the BIM model could be applied by a construction unit [56]. Therefore, changing the current project management mode is an important impetus to promote the development of BIM technology in tunnel engineering in China.

#### 4.1.4. Integration with GIS

**Figure 9.**
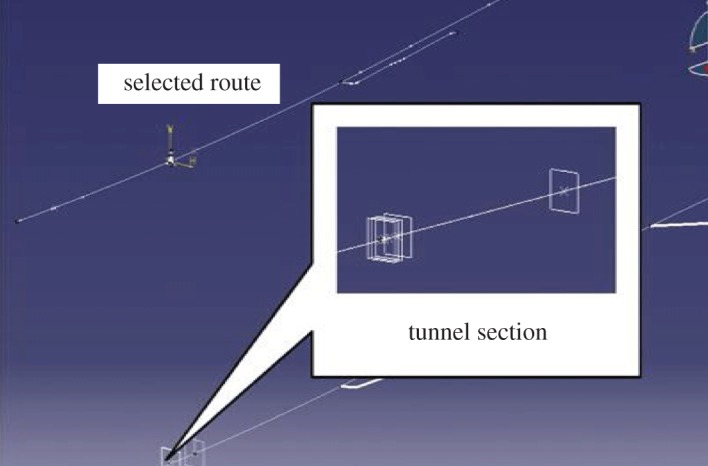
Selection of tunnel route.

Most tunnels are constructed in mountains, where complex geology and diversiform topography prevail. Therefore, it is relatively hard to apply BIM technology to tunnel engineering. GIS is a software technology focusing on geographic data [57]. If BIM technology is integrated with GIS, and an analysis about the BIM model based on GIS geographic data is conducted, the process of modelling and analysis will be greatly simplified [58–60]. Figure 9 presents the process of tunnel route selection based on GIS or a 3D geographical model, which provides great convenience for building information models in tunnels and rock masses. After finishing route selection, the model frame is determined. Cross-section sketches and parameters of different cross-sections of route will be surveyed, mapped and designed. Therefore, an effective integration of BIM and GIS is another task for the popularity of BIM technology in tunnel engineering in China.

#### 4.1.5. Poor awareness

At present, BIM technology is mainly applied for complex engineering regardless of general building or underground engineering. Generally, few units integrate applied BIM technology into design, which indicates insufficient understanding about BIM technology. BIM technology not only can shorten construction cycle and reduce construction cost; it also has significance for the whole life cycle of the project. BIM files, which are delivered to the operator after construction, will provide great convenience for later operation management and emergency.

Problems of standard, software platform and selection of project management mode are fundamental problems for both successful construction and BIM application. Additionally, the solution of these three problems will significantly promote BIM application in tunnel engineering in China.

### 4.2. Suggestions

The BIM standard system of tunnel engineering should be established. Achieving the advantages of BIM requires cooperation of all participants in the whole project cycle, for which information exchange between different software of designing, construction and operation units is necessary. However, due to different software manufacturers, information flow is hard to be achieved without a uniform standard, which will impede BIM in exerting advantages in the whole life cycle.

The development of professional BIM software for tunnel engineering should be promoted. Currently, the BIM project for tunnel engineering in China is mainly designed by architectural BIM software. However, BIM software should be developed that integrates the peculiarity and complexity of tunnel engineering. Moreover, compatibility of software should be prioritized; e.g. addressing the problem of compatibility between BIM and GIS will mean great progress can be achieved for the development of BIM in tunnel engineering in China.

A strong link should be formed between visual model and database. Three-dimensional or 4D models will undoubtedly enhance the visual enjoyment of the model, but at the same time it remains difficult to modify the model due to its complex spatial structure. However, this model-modifying process would become rather flexible if the link between visual model and database is set. Furthermore, this is important to promote the development of BIM technology in the tunnel field.

**Figure 10.**
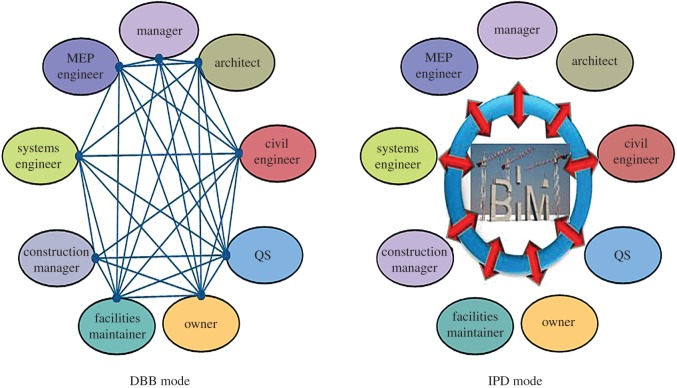
Application of IPD and DBB modes in tunnel engineering.

The traditional DBB management mode should be changed. IPD (Integrated Product Development) is a concept mode about product development, which now has been developed into a new management mode. Compared with the traditional DBB management mode, IPD emphasizes the collaboration ability and benefits of the whole project process. Different management modes create different information communication patterns; for DBB mode and IPD modes the differentiation of information communication patterns is shown in figure 10, which indicates that the DBB mode leads to a complex information environment, and wastes efforts on work collaboration and information synchronization, eventually reducing management efficiency. The application of the IPD mode will definitely simplify this complex information environment, reduce the cost of work collaboration and information synchronization, and eventually improve efficiency. The IPD management mode matches the requirements of BIM advantages. Hence, the reform of management mode becomes the most urgent matter for development of BIM technology in tunnel engineering in China.

Virtual Reality (VR) technology should be introduced into relative software. Generated BIM mode and various interface devices are applied to immerse users in a specified reality, which is convenient for the modification of BIM mode and operation management afterwards. Involving an environment of high-risk or extreme conditions, impossible and irreversible operation, large-scale or comprehensive training of high cost, VR technology can provide a relatively more reliable, safe and economic experimental project. For instance, tunnels propose strict requirements on emergency measures in the case of a fire disaster; therefore, the operation stage has high requirements for the expertise of emergency measures and personnel when a fire disaster occurs in a tunnel. VR technology allows for the construction of emergency rescue and safe training systems. It not only shows the scene of operation flow to personnel, instructing them to learn and master safe operation skills, but also simulates the scene of occurrence of disaster and fire, and enables personnel to simulate rescue in VR.

## 5. Concluding remarks

The development of BIM technology in tunnel engineering in China faces various problems, including a difference of standards, incompatibility of software, disorganized management, independence of GIS, low utilization of existing BIM modes and poor awareness, among which several factors including difference of standards, incompatibility of software and disorganized management are key problems hindering BIM development in tunnel engineering in China. The lack of integration with GIS is an urgent problem for BIM application in mountain tunnels, and problems of low utilization and poor awareness are induced by less training in BIM technology in tunnel engineering.

BIM technology is at the early stages in tunnel engineering in China, but China has already begun to introduce this technology into tunnel engineering, and it will gradually extend into the whole process of design, construction and operation stages.

Application of BIM technology in tunnel engineering integrates tunnel project, informatization and digitization, improves the collaboration of different units at various stages of construction, and greatly improves efficiency. During the design stage, statistics of quantities can be accomplished with sufficient precision. During the construction stage, resource allocation can be properly arranged, and construction simulation can be conducted. During the operation stage, a series of environmental conditions including fire disasters, emergency measures and convenience for operation management can be simulated, all of which would highly meet the requirements for environment protection. Therefore, BIM application in tunnel engineering will become an urgent trend.

Integration of BIM and tunnel engineering could introduce many benefits, while at the same time the challenge is great. To promote the development of BIM-based tunnel engineering in China, scholars should start from the formulation of a standard system, the development of relevant software, changing management mode and integration of BIM and GIS technologies, to accelerate the development of informatization and digitization in tunnel engineering in China.

## Data accessibility

BIM Data of the Shigu Mountain tunnel has been deposited at the website: http://www.shigongjishu.cn/Item/15084.aspx. BIM Data of the Xingu Mountain tunnel has been deposited at the website: http://www.beiweihy.com.cn/case/16034900.html.

## References

[1] Cerovsek T 2011 A review and outlook for a ‘building information model’ (BIM): a multi-standpoint framework for technological development. Adv. Eng. Inform. 25, 224–244. (doi:10.1016/j.aei.2010.06.003)

[2] He QH, Qian LL, Duan YF, Li Y-K 2012 Current situation and barriers of BIM implementation. J. Eng. Manage. 26, 12–16.

[3] Yalcinkaya M, Singh V 2015 Patterns and trends in building information modeling (BIM) research: a latent semantic analysis. Automation in Construction 59, 68–80. (doi:10.1016/j.autcon.2015.07.012)

[4] He GP 2011 Three pillars to realize the value of BIM IFC/IDMIFD. J. Info. Technol. Civil Eng. Arch. 3, 108–116.

[5] Liu HG 2015 Experience and enlightenment of application of BIM in abroad. High Speed Railway Technol. 6, 59–63.

[6] Li J, Wang Y, Wang XY, Luo H, Kang S-C, Wang J, Guo J, Jiao Y 2014 Benefits of building information modelling in the project lifecycle: construction projects in Asia. Int. J. Adv. Rob. Syst. 11, 1–2. (doi:10.5772/58447)

[7] Zheng GQ, Qiu KN 2012 Overview of building information modeling standard at home and broad. J. Info. Technol. Civil Eng. Arch. 4, 32–35.

[8] Lee S, Yu JH 2016 Comparative study of BIM acceptance between Korea and the United States. J. Construction Eng. Manage. 142, 1–9. (doi:10.1061/(ASCE)CO.1943-7862.0001076)

[9] Peng CH, Wu X 2015 Case study of carbon emissions from a building's life cycle based on BIM and Ecotect. Adv. Mater. Sci. Eng. 1, 1–15. (doi:10.1155/2015/954651)

[10] Gilkinson N, Raju P, Kiviniemi A, Chapman C 2015 Building information modelling: the tide is turning. Proc. Inst. Civil Eng. Struct. Buildings 168, 81–93. (doi:10.1680/stbu.12.00045)

[11] Asl MR, Zarrinmehr S, Bergin M, Yan W 2015 BPOpt: a framework for BIM-based performance optimization. Energy and Buildings 108, 401–412. (doi:10.1016/j.enbuild.2015.09.011)

[12] Liu H, Zhao J 2014 The analysis of resistances that hamper the use of BIM in China. Comput. Info. Technol. 519, 1447–1450. (doi:10.4028/www.scientific.net/AMM.519-520.1445)

[13] Shi YK 2014 BIM technology in the application and development of China's construction industry. Comput. Info. Technol. 519, 1451–1454. (doi:10.4028/www.scientific.net/AMM.519-520.1449)

[14] Liu H, Liu Q 2014 Research on the development barriers of BIM in China. Dev. Ind. Manufacturing 525, 691–694. (doi:10.4028/www.scientific.net/AMM.525.691)

[15] Zhou S, Huang ZH 2014 Analysis and research on the application status of BIM in China. J. Info. Technol. Civil Eng. Arch. 6, 24–29.

[16] Zhang SX, Hu YR 2014 The analysis of barriers of development of China's construction industry BIM. Struct. Environ. Eng. 838, 3119–3122. (doi:10.4028/www.scientific.net/AMR.838-841.3119)

[17] Cao DP, Wang GB, Li H, Skitmore M, Huang T, Zhang W 2015 Practices and effectiveness of building information modelling in construction projects in China. Automation in Construction 49, 113–122. (doi:10.1016/j.autcon.2014.10.014)

[18] Liu S, Meng XH, Tam C 2015 Building information modeling based on building design optimization for sustainability. Energy and Buildings 105, 139–153. (doi:10.1016/j.enbuild.2015.06.037)

[19] Zhu J 2010 Preliminary exploration on application of BIM in railway design. J. Railway Eng. Soc. 10, 104–108.

[20] Liu JT, Hu GC 2014 Research on application of BIM technology in railway design. High Speed Railway Technol. 5, 112–117.

[21] Shi YY, Lan T 2014 Analyses of the application for the BIM technology in railway construction. Railway Eng. Cost Manage. 29, 65–68.

[22] Li H, Guo HL, Huang T, Chan N, Chan G 2010 Research on the application architecture of BIM in building projects. J. Eng. Manage. 24, 525–529.

[23] Abanda FH, Vidalakis C, Oti AH, Tah JHM 2015 A critical analysis of building information modelling systems used in construction projects. Adv. Eng. Softw. 90, 183–201. (doi:10.1016/j.advengsoft.2015.08.009)

[24] Wong PF, Salleh H, Rahim FA 2015 A relationship framework for building information modeling (BIM) capability in quantity surveying practice and project performance. Informes De La Construction 67, 1–12. (doi:10.3989/ic.15.007)

[25] Lee S, Tae S, Roh S, Kim T 2015 Green template for life cycle assessment of buildings based on building information modeling: focus on embodied environmental impact. Sustainability 7, 16 498–16 512. (doi:10.3390/su71215830)

[26] Zhou JL, Wu YX, Yan XF 2014 The technology development of building information modeling in America and enlightenment towards building transformation in China. Sci. Technol. Progr. Policy 31, 30–33.

[27] Yang DL 2013 Summarize on current situation of foreign BIM implementation. J. Info. Technol. Civil Eng. Arch. 5, 89–94.

[28] He GP 2011 Development strategy and pattern of building information modeling in China (version 1). J. Info. Technol. Civil Eng. Arch. 3, 114–118.

[29] Wang ZJ, Ma AZ 2015 BIM technology and its application in the railway tunnel design. Construction Technol. 44, 59–63.

[30] Ryu DW, Kim JI, Suh S, Suh W 2015 Evaluating risks using simulated annealing and building information modeling. Appl. Math. Modell. 39, 5925–5935. (doi:10.1016/j.apm.2015.04.024)

[31] Marzouk M, Abdelaty A 2014 Monitoring thermal comfort in subways using building information modeling. Energy and Buildings. 84, 252–257. (doi:10.1016/j.enbuild.2014.08.006)

[32] Lai JX, Mao S, Qiu JL, Fan H, Zhang Q, Hu Z, Chen J 2016 Investigation progresses and applications of fractional derivative model in geotechnical engineering. Math. Problems Eng. 3, 1–15. (doi:10.18280/mmep.030101)

[33] Lai JX, Fan HB, Chen JX, Qiu J, Wang K 2015 Blasting vibration monitoring of undercrossing railway tunnel using wireless sensor network. Int. J. Distrib. Sens. Netw. 2, 1–7. (doi:10.1155/2015/703980)

[34] Wang JB, Liu XR, Song ZP, Guo JQ, Zhang QQ 2016 A creep constitutive model with variable parameters for thenardite. Environ. Earth Sci. 75, 1–12. (doi:10.1007/s12665-015-4873-x)

[35] Lai JX, Wang KY, Qiu JL, Niu F, Wang J, Chen J 2016 Vibration response characteristics of the cross tunnel structure. Shock and Vibration 5, 1–16. (doi: 10.1155/2016/9524206)

[36] Lai JX, Qiu JL, Chen JX, Wang YQ, Fan HB 2014 Application of wireless intelligent control system for HPS lamps and LEDs combined illumination in road tunnel. Comput. Intell. Neurosci. 2, 1–7. (doi:10.1155/2014/429657) 10.1155/2014/429657 PMC4281475 25587266

[37] Ruppel U, Schatz K 2011 Designing a BIM-based serious game for fire safety evacuation simulation. Adv. Eng. Info. 25, 600–611. (doi:10.1016/j.aei.2011.08.001)

[38] Kim K, Yu J 2014 BIM-based building energy load calculation system for designers. J. Civil Eng. 20, 549–563. (doi:10.1007/s12205-015-1625-0)

[39] Abanda FH, Byers L 2014 An investigation of the impact of building orientation on energy consumption in a domestic building using emerging BIM (Building Information Modelling). Energy 97, 517–527. (doi:10.1016/j.energy.2015.12.135)

[40] Lai JX, Qiu JL, Fan HB, Chen JX, Xie YL 2016 Freeze-proof method and test verification of a cold region tunnel employing electric heat tracing. Tunnell. Underground Space Technol. 60, 56–65. (doi:10.1016/j.tust.2016.08.002)

[41] Lai JX, Qiu JL, Feng ZH, Chen JX, Fan HB 2016 Prediction of soil deformation in tunnelling using artificial neural networks. Comput. Intell. Neurosci. 8, 1–16. (doi:10.1155/2016/6708183) 10.1155/2016/6708183 PMC4706869 26819587

[42] Xue WJ, Wang LB, Wang D, Druta C 2014 Pavement health monitoring system based on embedded sensing network. J. Mater. Civil Eng. 26, 1–8. (doi:10.1061/(ASCE)MT.1943-5533.0000778)

[43] Lai JX, Liu HQ, Qiu JL, Chen J 2016 Settlement analysis of saturated tailings dam treated by CFG pile composite foundation. Adv. Mater. Sci. Eng. 6, 1–10. (doi:10.1155/2016/7383762)

[44] Lai JX, Qiu JL, Fan HB, Zhang Q, Hu Z, Wang J, Chen J 2016 Fiber brag grating sensors-based in-situ monitoring and safety assessment of loess tunnel. J. Sensors 16, 1–10. (doi:10.1109/JSEN.2016.2616227)

[45] Luo YB, Chen JX, Xi WZ, Zhao PY, Qiao X, Deng XH, Liu Q 2016 Analysis of tunnel displacement accuracy with total station. Measurement 83, 29–37. (doi:10.1016/j.measurement.2016.01.025)

[46] Luo YB, Chen JX, Huang P, Tang MQ, Qiao X, Liu Q 2017 Deformation and mechanical model of temporary support sidewall in tunnel cutting partial section. Tunnell. Underground Space Technol. 61, 40–49. (doi:10.1016/j.tust.2016.09.007)

[47] Dai LF 2015 Study on current applications and problems of BIM in tunnel engineering. Railway Stand. Design 59, 99–103.

[48] Xu B 2015 Application research of BIM technology in Qingliang tunnel. Railway Technol. Innov. 3, 90–93.

[49] Li JS, Ye MZ, Zhao L 2015 The application of railway EBS decomposed system in design of Benzhong tunnel. Railway Technol. Innov. 3, 41–43.

[50] Cheng J 2014 Applications of BIM technology in the rail transportation engineering design. Chinese J. Underground Space Eng. 10, 1663–1668.

[51] Lai JX, Liu HQ, Qiu JL, Fan H, Zhang Q, Hu Z, Wnag J 2016 Stress analysis of CFG pile composite foundation in consolidating saturated mine tailings dam. Adv. Mater. Sci. Eng. 2, 1–12. (doi:10.1155/2016/3948754)

[52] He GP 2011 Development strategy and pattern of building information modeling in China (version 3). J. Info. Technol. Civil Eng. Arch. 3, 112–117.

[53] He GP 2011 Development strategy and pattern of building information modeling in China (version 2). J. Info. Technol. Civil Eng. Arch. 3, 112–117.

[54] Zhang JX 2010 Study on barriers of implementing BIM in engineering design Industry in China. J. Eng. Manage. 8, 387–392.

[55] Zhang LY, Li YW, Gao Y 2013 Barrier and countermeasures of the BIM application in China. J. Civil Eng. Manage. 3, 65–69.

[56] Xu JQ, Lu HM 2011 Information flow model for construction supply chain based on BIM. J. Eng. Manage. 25, 138–142.

[57] Qian Y 2013 Application discussion of combination of BIM and GIS in the cycle of rail-transit. Underground Eng. Tunnels 3, 40–43.

[58] Li DC, Zhang RZ 2012 Research on application of BIM technology in 3D modeling of digital city. J. Info. Technol. Civil Eng. Arch. 4, 47–51.

[59] Borrmann A, Kolbe TH, Donaubauer A, Steuer H, Jubierre JR, Flurl M 2015 Multi-scale geometric-semantic modeling of shield tunnels for GIS and BIM applications. Computer-aided Civil Infrastructure Eng. 30, 263–281. (doi:10.1111/mice.12090)

[60] Lai JX et al. In press. A state-of-the-art review of sustainable energy based freeze proof technology for cold-region tunnels in China. Renewable Sustainable Energy Rev.

